# Supplementary material for: First Isolation of Pseudogymnoascus destructans, the Fungal Causative Agent of White-Nose Syndrome, in Korean Bats (Myotis petax)
Source: J Fungi (Basel). 2022 Oct 12;8(10):1072. doi: 10.3390/jof8101072 (PMC9605074; doi:10.3390/jof8101072)
Supplement: Supplementary file 1 [file jof-08-01072-s001.zip › jof-1950594-supplementary.pdf]

Table S1. Captured bats information.

No	Bat Species	Sex	Province	City
1	Rhinolophus ferrumequinum	M	Chungcheongnam-do	Gongju
2	Rhinolophus ferrumequinum	F	Chungcheongnam-do	Gongju
3	Myotis petax	M	Chungcheongnam-do	Gongju
4	Myotis petax	M	Chungcheongnam-do	Gongju
5	Myotis aurascens	F	Chungcheongnam-do	Gongju
6	Myotis aurascens	M	Chungcheongnam-do	Gongju
7	Myotis aurascens	F	Chungcheongnam-do	Gongju
8	Myotis aurascens	F	Chungcheongnam-do	Gongju
9	Hypsugo alaschanicus	M	Chungcheongnam-do	Gongju
10	Hypsugo alaschanicus	F	Chungcheongnam-do	Gongju
11	Eptesicus serotinus	M	Chungcheongnam-do	Gongju
12	Pipistrellus abramus	F	Chungcheongnam-do	Gongju
13	Pipistrellus abramus	F	Chungcheongnam-do	Gongju
14	Pipistrellus abramus	F	Chungcheongnam-do	Gongju
15	Pipistrellus abramus	U	Chungcheongnam-do	Gongju
16	Pipistrellus abramus	M	Chungcheongnam-do	Gongju
17	Pipistrellus abramus	M	Chungcheongnam-do	Gongju
18	Hypsugo alaschanicus	F	Chungcheongnam-do	Gongju
19	Eptesicus serotinus	F	Jeollabuk-do	Imsil
20	Eptesicus serotinus	F	Jeollabuk-do	Imsil
21	Eptesicus serotinus	F	Jeollabuk-do	Imsil
22	Myotis aurascens	M	Jeollabuk-do	Imsil
23	Rhinolophus ferrumequinum	M	Jeollabuk-do	Imsil
24	Eptesicus serotinus	F	Jeollabuk-do	Jangsu
25	Pipistrellus abramus	F	Jeollabuk-do	Jangsu
26	Pipistrellus abramus	M	Jeollabuk-do	Jangsu
27	Pipistrellus abramus	F	Jeollabuk-do	Jangsu
28	Pipistrellus abramus	F	Jeollabuk-do	Jangsu
29	Pipistrellus abramus	M	Jeollabuk-do	Jangsu
30	Pipistrellus abramus	F	Jeollabuk-do	Jangsu
31	Pipistrellus abramus	M	Jeollabuk-do	Jangsu
32	Pipistrellus abramus	F	Jeollabuk-do	Jangsu
33	Eptesicus serotinus	M	Gyeongsangbuk-do	Yeongyang
34	Eptesicus serotinus	F	Gyeongsangbuk-do	Yeongyang
35	Eptesicus serotinus	F	Gyeongsangbuk-do	Yeongyang
36	Eptesicus serotinus	F	Gyeongsangbuk-do	Yeongyang
37	Eptesicus serotinus	F	Gyeongsangbuk-do	Yeongyang
38	Eptesicus serotinus	F	Gyeongsangbuk-do	Yeongyang
39	Eptesicus serotinus	F	Gyeongsangbuk-do	Yeongyang
40	Eptesicus serotinus	F	Gyeongsangbuk-do	Yeongyang
41	Myotis aurascens	M	Gyeongsangbuk-do	Yeongyang
42	Myotis aurascens	M	Gyeongsangbuk-do	Yeongyang
43	Myotis aurascens	M	Gyeongsangbuk-do	Yeongyang
44	Myotis petax	F	Gyeongsangbuk-do	Goryeong
45	Myotis petax	F	Gyeongsangbuk-do	Goryeong
46	Myotis petax	M	Gyeongsangbuk-do	Goryeong
47	Myotis petax	F	Gyeongsangbuk-do	Goryeong
48	Myotis petax	M	Gyeongsangbuk-do	Goryeong
49	Myotis petax	M	Gyeongsangbuk-do	Goryeong
50	Myotis petax	F	Gyeongsangbuk-do	Goryeong
51	Myotis petax	F	Gyeongsangbuk-do	Goryeong
52	Myotis petax	F	Gyeongsangbuk-do	Goryeong
53	Myotis petax	F	Gyeongsangbuk-do	Goryeong
54	Myotis petax	F	Gyeongsangbuk-do	Goryeong
55	Myotis petax	M	Gyeongsangbuk-do	Goryeong
56	Myotis petax	M	Gyeongsangbuk-do	Goryeong
57	Rhinolophus ferrumequinum	F	Gyeongsangbuk-do	Goryeong
58	Miniopterus schreibersi	M	Gyeongsangnam-do	Hapcheon
59	Myotis petax	F	Gyeongsangnam-do	Hapcheon

60	Myotis petax	F	Gyeongsangnam-do	Hapcheon
61	Myotis petax	F	Gyeongsangnam-do	Hapcheon
62	Myotis petax	M	Gyeongsangnam-do	Hapcheon
63	Rhinolophus ferrumequinum	F	Gyeongsangnam-do	Hapcheon
64	Myotis petax	M	Gyeongsangbuk-do	Yeongyang
65	Myotis petax	F	Gyeongsangbuk-do	Yeongyang
66	Myotis petax	M	Gyeongsangbuk-do	Yeongyang
67	Rhinolophus ferrumequinum	M	Gyeongsangbuk-do	Yeongyang
68	Myotis aurascens	F	Gyeongsangnam-do	Hapcheon
69	Pipistrellus abramus	M	Gyeongsangnam-do	Hapcheon
70	Pipistrellus abramus	M	Gyeongsangnam-do	Hapcheon
71	Pipistrellus abramus	F	Gyeongsangnam-do	Hapcheon
72	Pipistrellus abramus	F	Gyeongsangnam-do	Hapcheon
73	Myotis petax	M	Gyeongsangnam-do	Sancheong
74	Myotis petax	M	Gyeongsangnam-do	Sancheong
75	Myotis petax	M	Gyeongsangnam-do	Sancheong
76	Myotis petax	M	Gyeongsangnam-do	Sancheong
77	Myotis petax	F	Gyeongsangnam-do	Sancheong
78	Myotis petax	M	Gyeongsangnam-do	Sancheong
79	Myotis petax	F	Gyeongsangnam-do	Sancheong
80	Myotis petax	M	Gyeongsangnam-do	Sancheong
81	Myotis petax	M	Gyeongsangnam-do	Sancheong
82	Myotis petax	M	Gyeongsangnam-do	Sancheong
83	Myotis petax	M	Gyeongsangnam-do	Sancheong
84	Miniopterus schreibersi	F	Gyeongsangnam-do	Sancheong
85	Miniopterus schreibersi	M	Gyeongsangnam-do	Sancheong
86	Myotis petax	M	Gyeongsangnam-do	Hapcheon
87	Myotis petax	F	Gyeongsangnam-do	Hapcheon
88	Miniopterus schreibersi	M	Gyeongsangnam-do	Hapcheon
89	Miniopterus schreibersi	F	Gyeongsangnam-do	Hapcheon
90	Miniopterus schreibersi	F	Gyeongsangnam-do	Hapcheon
91	Miniopterus schreibersi	F	Gyeongsangnam-do	Hapcheon
92	Rhinolophus ferrumequinum	F	Gyeongsangnam-do	Hapcheon
93	Myotis petax	M	Gyeongsangbuk-do	Goryeong
94	Myotis petax	M	Gyeongsangbuk-do	Goryeong
95	Myotis petax	F	Gyeongsangbuk-do	Goryeong
96	Rhinolophus ferrumequinum	F	Gyeongsangbuk-do	Goryeong
97	Pipistrellus abramus	F	Gyeonggi-do	Pocheon
98	Pipistrellus abramus	M	Gyeonggi-do	Pocheon
99	Myotis aurascens	M	Gyeonggi-do	Pocheon
100	Hypsugo alaschanicus	M	Gyeonggi-do	Pocheon
101	Hypsugo alaschanicus	M	Gyeonggi-do	Pocheon
102	Pipistrellus abramus	M	Gyeonggi-do	Pocheon
103	Hypsugo alaschanicus	M	Gyeonggi-do	Pocheon
104	Hypsugo alaschanicus	M	Gyeonggi-do	Pocheon
105	Myotis aurascens	M	Gyeonggi-do	Pocheon
106	Eptesicus serotinus	F	Gyeonggi-do	Pocheon
107	Pipistrellus abramus	M	Gyeonggi-do	Pocheon
108	Pipistrellus abramus	M	Gyeonggi-do	Pocheon
109	Hypsugo alaschanicus	M	Gyeonggi-do	Pocheon
110	Hypsugo alaschanicus	F	Gyeonggi-do	Pocheon
111	Pipistrellus abramus	F	Gyeongsangbuk-do	Gunwi
112	Pipistrellus abramus	M	Gyeongsangbuk-do	Gunwi
113	Myotis aurascens	M	Gyeongsangnam-do	Hadong
114	Hypsugo alaschanicus	F	Gyeongsangnam-do	Hadong
115	Pipistrellus abramus	M	Gyeongsangnam-do	Hadong
116	Pipistrellus abramus	M	Gyeongsangnam-do	Hadong
117	Miniopterus schreibersi	M	Jeollabuk-do	Imsil
118	Miniopterus schreibersi	F	Jeollabuk-do	Imsil
119	Miniopterus schreibersi	U	Jeollabuk-do	Imsil
120	Rhinolophus ferrumequinum	M	Jeollabuk-do	Imsil

121	Miniopterus schreibersi	F	Jeollabuk-do	Imsil
122	Miniopterus schreibersi	F	Jeollabuk-do	Imsil
123	Miniopterus schreibersi	F	Jeollabuk-do	Imsil
124	Miniopterus schreibersi	F	Jeollabuk-do	Imsil
125	Miniopterus schreibersi	F	Jeollabuk-do	Imsil
126	Miniopterus schreibersi	F	Jeollabuk-do	Imsil
127	Miniopterus schreibersi	F	Gangwon-do	Hwacheon
128	Miniopterus schreibersi	F	Gangwon-do	Hwacheon
129	Miniopterus schreibersi	F	Gangwon-do	Hwacheon
130	Miniopterus schreibersi	F	Gangwon-do	Hwacheon
131	Miniopterus schreibersi	F	Gangwon-do	Hwacheon
132	Miniopterus schreibersi	F	Gangwon-do	Hwacheon
133	Rhinolophus ferrumequinum	F	Gangwon-do	Hwacheon
134	Miniopterus schreibersi	M	Gangwon-do	Hwacheon
135	Miniopterus schreibersi	F	Gangwon-do	Hwacheon
136	Miniopterus schreibersi	F	Gangwon-do	Hwacheon
137	Miniopterus schreibersi	M	Gangwon-do	Hwacheon
138	Miniopterus schreibersi	F	Gangwon-do	Hwacheon
139	Miniopterus schreibersi	F	Gangwon-do	Inje
140	Miniopterus schreibersi	F	Gangwon-do	Inje
141	Miniopterus schreibersi	F	Gangwon-do	Inje
142	Rhinolophus ferrumequinum	F	Gangwon-do	Inje
143	Miniopterus schreibersi	F	Gangwon-do	Inje
144	Rhinolophus ferrumequinum	F	Gangwon-do	Inje
145	Miniopterus schreibersi	F	Gangwon-do	Inje
146	Miniopterus schreibersi	M	Gangwon-do	Inje
147	Rhinolophus ferrumequinum	M	Gangwon-do	Inje
148	Miniopterus schreibersi	F	Jeollabuk-do	Jangsu
149	Miniopterus schreibersi	U	Jeollabuk-do	Jangsu
150	Murina leucogaster	F	Jeollabuk-do	Jangsu
151	Rhinolophus ferrumequinum	M	Gangwon-do	Hwacheon
152	Myotis macrodactylus	M	Gangwon-do	Hwacheon
153	Myotis macrodactylus	M	Gangwon-do	Hwacheon
154	Myotis macrodactylus	M	Gangwon-do	Hwacheon
155	Myotis macrodactylus	M	Gangwon-do	Hwacheon
156	Myotis macrodactylus	M	Gangwon-do	Hwacheon
157	Myotis macrodactylus	M	Gangwon-do	Hwacheon
158	Rhinolophus ferrumequinum	F	Gangwon-do	Hwacheon
159	Myotis macrodactylus	M	Gangwon-do	Inje
160	Myotis macrodactylus	M	Gangwon-do	Inje
161	Myotis macrodactylus	M	Gangwon-do	Inje
162	Myotis macrodactylus	M	Gangwon-do	Inje
163	Myotis macrodactylus	M	Gangwon-do	Inje
164	Myotis macrodactylus	M	Gangwon-do	Inje
165	Myotis macrodactylus	M	Gangwon-do	Inje
166	Myotis macrodactylus	M	Gangwon-do	Inje
167	Myotis macrodactylus	M	Gangwon-do	Inje
168	Myotis macrodactylus	M	Gangwon-do	Inje
169	Rhinolophus ferrumequinum	M	Gangwon-do	Inje
170	Myotis macrodactylus	M	Jeollabuk-do	Jangsu
171	Myotis macrodactylus	M	Jeollabuk-do	Jangsu
172	Myotis macrodactylus	M	Jeollabuk-do	Jangsu
173	Myotis macrodactylus	M	Jeollabuk-do	Jangsu
174	Rhinolophus ferrumequinum	F	Jeollabuk-do	Jangsu
175	Miniopterus schreibersi	M	Jeollabuk-do	Jangsu
176	Rhinolophus ferrumequinum	F	Jeollabuk-do	Jangsu
177	Rhinolophus ferrumequinum	F	Jeollabuk-do	Jangsu
178	Myotis macrodactylus	M	Jeollabuk-do	Muju
179	Myotis macrodactylus	M	Jeollabuk-do	Muju
180	Myotis macrodactylus	M	Jeollabuk-do	Muju
181	Myotis macrodactylus	M	Jeollabuk-do	Muju

182	Myotis macrodactylus	M	Jeollabuk-do	Muju
183	Miniopterus schreibersi	M	Jeollabuk-do	Muju
184	Miniopterus schreibersi	M	Jeollabuk-do	Muju
185	Myotis macrodactylus	M	Jeollabuk-do	Muju
186	Myotis macrodactylus	M	Jeollabuk-do	Muju
187	Myotis macrodactylus	M	Jeollabuk-do	Muju
188	Myotis macrodactylus	M	Jeollabuk-do	Muju
189	Myotis macrodactylus	M	Jeollabuk-do	Muju
190	Myotis macrodactylus	M	Jeollabuk-do	Muju
191	Rhinolophus ferrumequinum	F	Jeollabuk-do	Muju
192	Rhinolophus ferrumequinum	F	Jeollabuk-do	Muju
193	Rhinolophus ferrumequinum	F	Jeollabuk-do	Muju
194	Rhinolophus ferrumequinum	F	Jeollabuk-do	Imsil
195	Rhinolophus ferrumequinum	F	Jeollabuk-do	Imsil
196	Rhinolophus ferrumequinum	F	Jeollabuk-do	Imsil
197	Rhinolophus ferrumequinum	F	Jeollabuk-do	Imsil
198	Miniopterus schreibersi	F	Jeollabuk-do	Imsil
199	Rhinolophus ferrumequinum	F	Gyeongsangnam-do	Sancheong
200	Rhinolophus ferrumequinum	F	Gyeongsangnam-do	Sancheong
201	Rhinolophus ferrumequinum	F	Gyeongsangnam-do	Sancheong
202	Rhinolophus ferrumequinum	M	Gyeongsangnam-do	Sancheong
203	Rhinolophus ferrumequinum	F	Gyeongsangnam-do	Sancheong
204	Miniopterus schreibersi	M	Gyeongsangnam-do	Sancheong
205	Miniopterus schreibersi	F	Gyeongsangnam-do	Sancheong
206	Rhinolophus ferrumequinum	M	Gyeongsangnam-do	Yeongyang
207	Rhinolophus ferrumequinum	M	Gyeongsangnam-do	Yeongyang
208	Rhinolophus ferrumequinum	M	Gyeongsangnam-do	Yeongyang
209	Rhinolophus ferrumequinum	F	Gyeongsangnam-do	Yeongyang
210	Rhinolophus ferrumequinum	F	Gyeongsangnam-do	Yeongyang
211	Rhinolophus ferrumequinum	M	Gyeonggi-do	Pocheon
212	Rhinolophus ferrumequinum	M	Gyeonggi-do	Pocheon
213	Rhinolophus ferrumequinum	M	Gyeonggi-do	Pocheon
214	Rhinolophus ferrumequinum	M	Gyeonggi-do	Pocheon
215	Rhinolophus ferrumequinum	M	Gyeonggi-do	Pocheon
216	Rhinolophus ferrumequinum	F	Gyeongsangnam-do	Hapcheon
217	Rhinolophus ferrumequinum	M	Gyeongsangnam-do	Hapcheon
218	Rhinolophus ferrumequinum	M	Gyeongsangbuk-do	Goryeong
219	Rhinolophus ferrumequinum	M	Gyeongsangbuk-do	Goryeong
220	Miniopterus schreibersi	M	Jeollabuk-do	Muju
221	Rhinolophus ferrumequinum	M	Chungcheongnam-do	Gongju
222	Rhinolophus ferrumequinum	M	Chungcheongnam-do	Gongju
223	Miniopterus schreibersi	M	Jeollabuk-do	Muju
224	Rhinolophus ferrumequinum	M	Jeollabuk-do	Muju
225	Rhinolophus ferrumequinum	F	Jeollabuk-do	Muju
226	Rhinolophus ferrumequinum	F	Gyeongsangnam-do	Hadong
227	Rhinolophus ferrumequinum	F	Gyeongsangnam-do	Hadong
228	Rhinolophus ferrumequinum	F	Gyeongsangnam-do	Hadong
229	Rhinolophus ferrumequinum	M	Gyeongsangnam-do	Hadong
230	Rhinolophus ferrumequinum	M	Gyeongsangnam-do	Hadong
231	Rhinolophus ferrumequinum	F	Gyeongsangnam-do	Hadong
232	Rhinolophus ferrumequinum	F	Gyeongsangnam-do	Hadong
233	Miniopterus schreibersi	F	Gyeongsangnam-do	Hadong
234	Miniopterus schreibersi	F	Gyeongsangnam-do	Hadong
235	Rhinolophus ferrumequinum	F	Gyeongsangnam-do	Hadong
236	Rhinolophus ferrumequinum	F	Gyeongsangnam-do	Hadong
237	Rhinolophus ferrumequinum	M	Gyeongsangnam-do	Hadong
238	Rhinolophus ferrumequinum	F	Gyeongsangnam-do	Hadong
239	Rhinolophus ferrumequinum	F	Gyeongsangnam-do	Hadong
240	Miniopterus schreibersi	M	Gyeongsangnam-do	Hadong
241	Miniopterus schreibersi	M	Gyeongsangnam-do	Hadong

Table S2. GenBank accession numbers of the sequences used in this study. Sequences highlighted in bold were generated in this study [1-4].

No	Species	Strain	Locality	Substrate /Host	Accession No. ITS	LSU	MCM7	RPB2	TEF1
1	*Pseudogymnoascus destructans*	20631-21 (ex-type)	USA, New York	*Myotis lucifugus*	EU884921	KF017865	KF017691	KF017747	KF017806
2	*Geomyces auratus*	CBS 108.14	Norway	Soil, filter paper bait	KF039895	KF017864	KF017690	KF017746	KF017805
3	*Leuconeurospora pulcherrima*	CBS 343.76	Switzerland	Forest Soil	KF049206	FJ176884	na	FJ238367	FJ238409
4	*Pseudeurotium zonatum*	CBS 329.36/ AFTOL-ID 1912	France	Soil near gas leakage	AY129286	DQ470988	na	DQ470940	DQ471112
5	*Pseudogymnoascus roseus*	03VT05	USA, Vermont	Hibernacular soil	KF039892	KF017820	KF017651	KF017705	KF017760
6	*Pseudogymnoascus roseus*	05NY06	USA, New York	Hibernacular soil	JX270385	KF017824	KF017655	KF017709	KF017764
7	*Pseudogymnoascus roseus*	05NY08	USA, New York	Hibernacular soil	JX270387	KF017825	KF017656	KF017710	KF017765
8	*Pseudogymnoascus palmeri*	LHU407	USA, Pennsylvania	Cave sediment	MT988150	na	na	MW054468	MW054467
9	*Pseudogymnoascus palmeri*	WSF 3629	USA, Wisconsin	Amorphus peat	KF039897	KF017870	KF017696	KF017751	KF017811
9	*Pseudogymnoascus turneri*	LHU121	USA, Pennsylvania	Sediment	MN542213	na	na	MN541380	MN541379
10	*Pseudogymnoascus turneri*	Ps5	USA, Pennsylvania	Sediment	MN542214	na	na	MN541382	MN541381
11	*Pseudogymnoascus turneri*	23342-1-I1	USA, Wisconsin	*Perimyotis subflavus*	JX415266	KF017868	KF017694	KF017749	KF017809
12	*Pseudogymnoascus lindneri*	02NH05	USA, New Hampshire	Hibernacular soil	JX270350	KF017818	KF017649	KF017703	KF017758
13	*Pseudogymnoascus lindneri*	LHU158	USA, Pennsylvania	Sediment	MN542212	na	na	MN541384	MN541383
14	*Pseudogymnoascus appendiculatus*	02NH11	USA, New Hampshire	Hibernacular soil	JX270356	KF017819	KF017650	KF017704	KF017759
15	*Pseudogymnoascus appendiculatus*	07MA02	USA, Massachusetts	Hibernacular soil	JX270402	KF017827	KF017658	KF017712	KF017767
16	*Pseudogymnoascus pannorum*	ATCC 16222	Germany, West Germany	Soil from wheat field	AF015789	na	KF686777	na	KF686766
17	*Pseudogymnoascus shaanxiensis*	GZUIFR HZ5.7	China	Soil	MT509366	MT509380	MT534206	MT534220	MT534231
18	*Pseudogymnoascus yunnanensis*	GZUIFR 21.807	China	Soil	MZ444072	MZ444099	MZ490754	MZ488537	MZ488514
19	*Pseudogymnoascus yunnanensis*	GZUIFR 21.808	China	Soil	MZ444073	MZ444100	MZ490755	MZ488538	MZ488515
20	*Pseudogymnoascus zhejiangensis*	GZUIFR 21.810	China	Soil	MZ444075	MZ444102	MZ490757	MZ488540	MZ488517
21	*Pseudogymnoascus zhejiangensis*	GZUIFR 21.811	China	Soil	MZ444076	MZ444103	MZ490758	MZ488541	MZ488518
22	*Pseudogymnoascus catenatus*	GZUIFR 21.813	China	Soil	MZ444078	MZ444105	MZ490760	MZ488543	MZ488520
23	*Pseudogymnoascus catenatus*	GZUIFR 21.814	China	Soil	MZ444079	MZ444106	MZ490761	MZ488544	MZ488521
24	*Pseudogymnoascus fujianensis*	GZUIFR 21.819	China	Soil	MZ444084	MZ444111	MZ490766	MZ488549	MZ488526
25	*Pseudogymnoascus fujianensis*	GZUIFR 21.820	China	Soil	MZ444085	MZ444112	MZ490767	MZ488550	MZ488527
26	*Pseudogymnoascus guizhouensis*	GZUIFR 376.1	China	Soil	MT509369	MT509383	MT534209	MT534223	MT534234
27	*Pseudogymnoascus guizhouensis*	GZUIFR 376.2	China	Soil	MT509370	MT509384	MT534210	MT534224	MT534235
28	*Pseudogymnoascus sinensis*	CGMCC 3.18493(K278)	China	Soil	MT509364	MT509378	MT534204	MT534218	MT534229
29	*Pseudogymnoascus verrucosus*	VKM F-103	USA, New York	Top soil	JPKB01001524<br>(1 to 491)	JPKB01001524<br>(560 to 1413)	JPKB01001753<br>(13559 to 14177)	JPKB01001744<br>(6669 to 7432)	JPKB01001786<br>(12082-12985)
30	*Pseudogymnoascus verrucosus*	UAMH 10579	Canada, Alberta	brown-rotted wood bait block	NR_111197	na	XM_018270150	XM_018270703	XM_018277618
31	*Pseudogymnoascus antarcticus*	CHFC-EA 569(F09-T2-1)	Antarctica	Antarctic marine sponge	JX845280	MN417282	MN432493	MN418135	MN418131

No	Species	Strain	Locality	Substrate /Host	Accession No.				
					ITS	LSU	MCM7	RPB2	TEF1
32	*Pseudogymnoascus australis*	CHFC-EA 567(F09-T18-3)	Antarctica	Antarctic marine sponge	MN417287	MN417284	MN432491	MN418137	MN418133
33	*Pseudogymnoascus griseus*	CHFC-EA 568(F09-T18-14)	Antarctica	Antarctic marine sponge	MN417288	MN417285	MN432492	MN418138	MN418134
34	*Pseudogymnoascus lanuginosus*	CHFC-EA 570(F09-T18-27)	Antarctica	Antarctic marine sponge	MN417286	MN417283	MN418139	MN418136	MN418132
35	*Pseudogymnoascus pannorum*	ATCC 16222	Germany, West Germany	Soil from wheat field	AF015789	na	KF686777	na	KF686766
36	*Pseudogymnoascus destructans*	BW48	South Korea	Myotis petax	OP420747	OP420753	OP450957	OP450959	OP450961
37	*Pseudogymnoascus destructans*	BW49	South Korea	Myotis petax	OP420748	OP420754	OP450958	OP450960	OP450962

References

1. Minnis, A.M.; Lindner, D.L. Phylogenetic evaluation of Geomyces and allies reveals no close relatives of Pseudogymnoascus destructans, comb. nov., in bat hibernacula of eastern North America. *Fungal Biology* **2013**, *117*, 638–649, doi:10.1016/j.funbio.2013.07.001.
2. Villanueva, P.; Vásquez, G.; Gil-Durán, C.; Oliva, V.; Díaz, A.; Henríquez, M.; Álvarez, E.; Laich, F.; Chávez, R.; Vaca, I. Description of the First Four Species of the Genus Pseudogymnoascus From Antarctica. *Front Microbiol* **2021**, *12*, 713189, doi:10.3389/fmicb.2021.713189.
3. Zhang, Z.; Dong, C.; Chen, W.; Mou, Q.; Lu, X.; Han, Y.; Huang, J.; Liang, Z. The Enigmatic Thelebolaceae (Thelebolales, Leotiomycetes): One New Genus Solomyces and Five New Species. *Front Microbiol* **2020**, *11*, 572596, doi:10.3389/fmicb.2020.572596.
4. Zhang, Z.Y.; Shao, Q.Y.; Li, X.; Chen, W.H.; Liang, J.D.; Han, Y.-F.; Huang, J.-Z.; Liang, Z.-Q. Culturable Fungi from Urban Soils in China I: Description of 10 New Taxa. *Microbiology Spectrum* **2021**, *9*, e00867–00821, doi:10.1128/Spectrum.00867-21.
